# Concussion Rehabilitation and the Application of Ten Movement Training Principles

**DOI:** 10.7759/cureus.46520

**Published:** 2023-10-05

**Authors:** James McLoughlin

**Affiliations:** 1 College of Nursing and Health Sciences, Flinders University, Adelaide, AUS

**Keywords:** training, movement, rehabilitation, traumatic brain injuries, concussion

## Abstract

Concussion awareness continues to grow in all aspects of healthcare, including the areas of prevention, acute care, and ongoing rehabilitation. Most of the concussion research to date has focussed on the challenges around screening and diagnosing what can be a complex mix of brain impairments that overlay with additional pre-existing comorbidities. While we expect further progress in concussion diagnosis, progress also continues to be made around proactive rehabilitation, with the emergence of interventions that can enhance the recovery process, maximise function and independence with a return to study, work, and play.

Traditionally, optimal multimodal assessments of concussion have treated the physical, cognitive, and psychological domains of brain injury separately, which supports diagnosis, and informs appropriate follow-up care. Due to the complex nature of brain injury, multimodal assessments direct care toward professionals from many different disciplines including medicine, physiotherapy, psychology, neuropsychology, ophthalmology, and exercise physiology. In addition, these professionals may work in different fields such as sports, neurorehabilitation, vestibular, musculoskeletal, community, vocational, and general practice clinical settings. Rehabilitation interventions for concussions employed in practice are also likely to use a blend of theoretical principles from motor control, cognitive, and psychological sciences. This scale of diversity can make information dissemination, collaboration, and innovation challenging. The Ten Movement Training Principles (MTPs) have been proposed as a usable and relevant concept to guide and support clinical reasoning in neurorehabilitation. When applied to concussion rehabilitation, these same 10 principles provide a comprehensive overview of key rehabilitation strategies for current and future practice. Future collaborations can use these training principles to support clinical and research innovations including the rapid rise of technologies in this growing field of rehabilitation practice.

## Introduction and background

Concussions are fairly common injuries. In the United States, between 1.7 and 3 million concussions occur each year, with 223,000 traumatic brain injury (TBI)-related hospitalisations [1]. In Australia, it is estimated that around 200,000 TBIs occur each year with around 180,000 of them likely to be mild TBIs/concussions [2]. In Europe, regional studies show an incidence rate of TBI up to 849 per 100,000 population per year [3]. Causes of concussion range from falls, motor vehicle accidents, domestic violence, and sporting and playground accidents.

Concussion diagnosis and recovery have become part of public discussion with growing awareness, particularly around elite athletes. Multimodal assessment and rehabilitation of concussion can be complex, with the potential for many different brain functions to be impaired. Multimodal assessments play an important role in early diagnostic assessments and as well as in examining those with persistent signs and symptoms. This includes physical, cognitive, and psychological changes that need to be assessed, monitored, and targeted as part of an optimal rehabilitation program. Many health professionals bring their expertise into the assessment and rehabilitation of concussion including medical, physiotherapy, psychology, neuropsychology, ophthalmology, and exercise physiology. In addition, these professionals may work in different clinical fields such as sports, neurorehabilitation, emergency, vestibular, community, vocational, and general practice settings. Most of the research literature concerning concussions comes from the sporting fields; however, concussion is a common occurrence outside of this field where it is frequently seen in emergency departments, school clinics, and general practice. Neurological and vestibular clinics also work with many people with concussion/TBI as part of best-practice neurorehabilitation, where multidisciplinary links are made.

Rapid progress in concussion assessment and rehabilitation over the past decade has been exciting, but it also presents challenges that arise when bringing such a diverse group of clinical and research expertise together. It is already known that progress in rehabilitation and training can be affected by confusion related to vague language [4]. Concussion interventions have evolved from many clinical fields including neurocognitive, musculoskeletal, sports, vestibular, visual, and psychology. If we are to make further progress, these fields will need to collaborate to explore promising research and disseminate and implement current best practices.

The Ten Movement Training Principles (MTPs) aim to help this process by providing consistent terminology and have been previously discussed in relation to neurorehabilitation [5]. This paper will discuss MTPs in relation to concussion rehabilitation, where each training principle will review training targets that are emerging in research and clinical practice. A review of concussion rehabilitation in relation to each MTP aims to provide a clear and comprehensive insight into the clinical reasoning process that informs individualised, multidisciplinary rehabilitation. The MTPs may potentially help in the design of future multimodal interventions that combine training principles that might then be enhanced with innovation and future technologies. Explicit and accessible information about proactive rehabilitation following concussion may also help with the current culture of under-reporting of concussions, improve symptom disclosure, and guide people toward the most appropriate health professionals. Table 1 provides a summary of the 10-MTPs with a focus on factors to consider in concussion rehabilitation.

**Table 1:** A summary of the Ten Movement Training Principles with factors to consider in concussion rehabilitation

Movement training principle	Factors to consider in concussion rehabilitation
Actual and predicted bodily state	Body schema. Spatial cognition. Cervical joint position sense errors
Feedback	Visual and vestibular feedback dependence. Sensory reweighting. Integration and perceptual dysfunction. Overemphasis on symptom reporting
Error-based learning	Gaze stability and balance training. Vision training
Reward-based learning	Behavioural changes. Motivations and pressures. Managing expectations
Cognitive selection and planning	Memory and concentration changes. Executive dysfunction. Cognitive adaptations, loads, and fatigue. Slowed processing speed. Difficulty with increasing complexity
Practice and variability	Time for learning and adaptation. Frequency, intensity, and duration of practice to be effective. Variability and integration of different domains into rehabilitation. Autonomic and cardiovascular limitations. Prescribed rests/breaks
Biomechanics	Forces for brain damage and cervical whiplash injury. Prevention via protective gear. Prevention via motor skill training. Motor control and muscle compensations. Post-traumatic benign paroxysmal positional vertigo (BPPV)
Physical capacity	Detrimental rest. Autonomic dysfunction and postural orthostatic tachycardia syndrome (POTS). Deconditioning. Neck strength and stability
Attentional focus	Dual-task challenges. Attention-deficit/hyperactivity disorder (ADHD) and concussion testing. Functional overlay, external focus, and distraction techniques. Overemphasis on symptoms and hypervigilance illness behaviours
Beliefs and self-efficacy	Anxiety. Depression. Post-traumatic stress disorder (PTSD). Psychological flexibility. Negative beliefs and nocebo effects. Community misinformation and messaging

## Review

Actual and predicted bodily state

Actual and predicted bodily state refers to the proprioceptive awareness of body segments in relation to each other, the external environment, and a spatial awareness of the surroundings. All these abilities can become dysfunctional following a concussion. A reduced proprioceptive awareness of neck position in relation to external space by using the cervical joint position sense error test has been described in some studies. This measure has been shown to increase concussion risk in rugby players and can easily be added to the cervical examination in general practice. Spatial awareness can also be affected, with a study of concussed adolescent hockey players demonstrating an impairment in the ability to form cognitive maps [6-9]. People who sustained a concussion more than a month prior demonstrated decision-making impairments in the ability to walk to a target via an obstacle with less cautious behaviour. A similar impaired judgement was observed in those with a history of concussion (in the past year) as they tried to avoid another walker by approaching from a 90-degree angle [10, 11]. In sports, subsequent injury risk following concussion is increased, but the types of injury and the type of sport may need to be considered more closely [12]. A large study of junior Australian rules footballers showed similar increased subsequent injury risk following both concussion and non-concussion injuries, with the concussion group less likely to sustain a contact-type injury [13]. This raises further questions about not only the mechanism of injuries but on-field behaviour and decision-making that may relate to spatial cognition for specific sports and day-to-day tasks.

Two types of reference frames constitute spatial cognition. The egocentric reference frame refers to the ability to locate objects in the environment in reference to self. The allocentric reference frame refers to the ability to locate objects in relation to each other, or another fixed landmark in the environment. While there are clinical tests that help measure cervical position sense, the challenge is to find and use other clinical tests that help measure the function of both reference frames of spatial cognition. Virtual reality tasks show promise in this area, where it has been used in Alzheimer’s disease and, more recently, concussions [14,9].

Training the ability to scan and search the visual world when moving, while holding a memory of spatial information may become a key part of concussion rehabilitation. Inaccurate perception seems to affect quick decision-making, which is likely to contribute to injury and repeat concussions in sports. Ongoing deficits may also influence the decision-making around readiness for return to work or play. An active process of learning via dynamic interaction with the environment can promote multisensory integration of visual, vestibular, and proprioceptive inputs in addition to allocentric and egocentric reference frames of spatial cognition [15].

Feedback

External and internal feedback are important tools used in rehabilitation. External feedback can be provided by therapists or coaches via verbal prompts, visual feedback tools such as laser pointers/mirrors, or via tactile cues. Internal feedback from visual, proprioceptive, and vestibular inputs also helps with motor learning and control. If feedback is to be perceived and integrated effectively, the sensory, attention, and integrational networks in the brain need to be effective. In terms of controlling standing balance, concussions can lead to an over-dependence on visual and vestibular feedback as indicated by exaggerated responses to changes in the visual scene or galvanic vestibular stimulation [16]. This means that sensory feedback about visual or vestibular motion results in reduced control of postural sway. This indicates a change in sensorimotor processing that would influence the adaptive processing that is exploited through sensory substitution in vestibular rehabilitation [15]. Vestibular rehabilitation is emerging as an effective early intervention following concussion to reduce dizziness and improve balance [17]. Further knowledge around sensory reweighting with increased visual dependency will help with the design and selection of individual types of training used.

Reduced cortical excitability following concussions also results in reduced ability to maximally activate muscle activity with additional change in perception of force [18]. If you combine this with a dependency on vision and the reduced ability to upregulate other sensory information for action, the selection and progression of movement training strategies will need to take this into account. This may require a more structured way of introducing different sensory inputs to enable the perception and control of movement to be gradually learned.

To date, the diagnosis of concussion and the decisions around recovery have been heavily determined by symptom reporting. Symptoms like headache, dizziness, visual blurring, feelings of fogginess, anxiety, nausea, sensitivity to light or noise, or problems with concentration are just some of the symptoms that form part of a continual assessment process. In rehabilitation, people with concussions are always asked for feedback on their symptoms. This can be important when designing sub-symptom aerobic exercise plans for example, where feedback around symptoms and levels of exertion are collected every minute during the Buffalo treadmill test [19]. However, as we discuss in the final principle of belief and self-efficacy, over-emphasising symptom feedback may create other problems around anxiety and low confidence, which can lead to delayed recovery. Symptom reporting and types of feedback used in concussion rehabilitation certainly require further investigation. A focus on symptom improvement and education around symptom types and expectations may be a sensible strategy, as well as developing further objective tests that measure signs of dysfunction and recovery that do not rely on symptom reporting.

Error-based learning

The feedforward predictive abilities of the brain allow expected sensory inputs to be compared to actual sensations. Any differences in what is predicted are referred to as sensory prediction errors, and these are important in many types of error-based learning. Error-based learning for gaze stability and balance strategies is likely to be fundamental for the effective visual and vestibular rehabilitation interventions used following concussions [17].

Vision training often targets convergence and accommodative insufficiency commonly seen after concussion [20,21], while gaze stability training is an integral part of vestibular rehabilitation where adaptation of the vestibular-ocular reflex uses error-based learning of retinal slip when the eyes stabilise on a target during incremental increases in head speeds. Studies have shown a critical period of enhanced neuroplasticity in this form of learning in the first few weeks following unilateral vestibular hypofunction insult [22], with relevance for concussion when considering that peripheral vestibular nerve dysfunction can occur [23]. This supports the role of early, specific vestibular rehabilitation to improve outcomes for a subset of concussions with peripheral vestibular signs.

Error-based learning is likely to be fundamental for skill acquisition in the rehabilitation of balance, vertigo, and vision. Strategies that consider types of sensory errors and the ways these can be amplified and manipulated to enhance learning in therapy following concussion need further research.

Reward-based learning

Reward-based learning is integral to rehabilitation as it relates to the deep-seated motivations that reinforce learning and behaviour. Rewards are likely influenced by individual and cultural factors, where the brain gives credit to priority outcomes that match the desires and needs of the individual. Reward-based learning is likely to help consolidate and retain important memories and skills that help with recovery [24,25].

Concussion brings a set of challenges that are likely to impact reward-based learning. One major issue is low levels of disclosure in sports, with one study showing that 22% of school students do not report concussions to an adult [26, 27]. Poor knowledge of the signs and symptoms of concussion and concerns about missing sporting games and employment opportunities are likely to not only lead to non-reporting of symptoms but also influence the motivations that drive people to seek out and engage in effective rehabilitation. The wider sporting culture should also be considered. Sports fans, particularly waging sports fans who exaggerate their concussion knowledge are likely to contribute to nondisclosure pressure in athletes [28].

Incentives help with the rehabilitation goal-setting process to drive motivation and can be useful when there is a desire to return to work or play. In sports, incentives for some may relate to returning to play earlier, while for others in general practice, it may be more about symptom relief. Therefore, goal-setting needs to be tailored to the individual. Premature return to play is a major concern with an increased risk of further concussion and a two-fold increase in musculoskeletal injury [29,12]. These health risks may motivate some to ensure optimal recovery time and training before return to play, while for others the risk of poor performance, loss of form, and confidence for an early return might be more motivating given that professional sport is performance-driven [30,31]. Delayed recovery with persistent symptoms influences behaviour, and hence objective tests of performance that demonstrate recovery and improvement should be included. Managing expectations is also critical as signs and symptoms will rapidly improve for many people within weeks, while persistent symptoms can occur in over 50% of people up to one-year post-concussion [32]. Symptoms such as headache, dizziness, apathy, and fatigue can all influence motivation and therefore reward-based learning. Care with the delivery of information following assessment is likely to be important, along with the use of strategies to build confidence in meaningful activities. Managing anxiety and stress is also important as this can interfere with the learning and compensation processes required for optimal recovery and cognitive performance [33].

Cognitive selection and planning

Cognitive processes such as memory, concentration, and attention can all be affected following concussion, and these are important factors to be considered within training programs. Executive function refers to the ability to organise external stimuli and plan a suitable response and involves working memory, self-control, and flexible thinking. It has been shown that executive function changes with tasks that involve conflict resolution and task switching following concussion [34]. For example, in a lower limb leg press task, brain activity in adolescents with a history of concussion shows that the left inferior parietal lobule/supramarginal gyrus (IPL) increases its activity and connectivity with the right posterior cingulate gyrus/precuneus cortex and right IPL [35]. This shift in brain networking may lead to movement compensations, increased neural drive, fatigue, and limitations with the demands of increasing complexity such as when additional dual tasks are included. This has been demonstrated in gait performance following concussions [36,37].

A visually guided motor task that requires additional cognitive-motor integration can identify concussion and is also present in asymptomatic, previously concussed elite athletes with slower reaction time and reduced accuracy [38,39]. More research is needed to understand the relationship between different neurocognitive functions and their role in injury risk in sports [40]. Processing speed within brain networks is likely to be important, with simple reaction time testing showing comparable sensitivity and specificity for detecting concussion when compared to more detailed and time-consuming neurocognitive testing protocols [41]. Faster reaction times have been shown to reduce the risk of severe head impact in American football [42] and ice hockey [43]. Simple reaction time tests can also be easily employed in general practice. These rapid perception and action functions should not only be part of the assessment but can also be introduced into rehabilitation programs [44]. Somatosensory reaction time and increased intracortical inhibition with transcranial magnetic stimulation have been demonstrated in those people with persistent symptoms following concussion [45]. Context is important in rehabilitation, with a need to move away from clinical and laboratory tests to task performance at a specific level in real-world scenarios. Training programs that gradually increase the complexity of cognitive performances required for specific tasks could involve incorporating expertise from occupational therapists for return to work and activities of daily living, and speciality coaches in the case of sports.

Practice and variability

Following brain injury, a person needs time to adapt and learn with practice. It is important to build confidence in home and work tasks, including sports and recreational activities. Practice with variability helps build capacity, such as strength and fitness, while specific exercises can target skill acquisition for tasks that are integral to recovery. Each type of practice will likely require different approaches in terms of frequency, intensity, and duration depending on the goals around capacity building, types of behavioural, cognitive, or motor learning, and symptom provocation. Variability within practice helps drive adaptation and learning that reflects real-world demands.

Vision training after concussion has been shown to be beneficial following 20 minutes of specific training, twice a week, for six weeks, or six times per week for 2.5 weeks with a once-per-week maintenance program [21]. Rehabilitation for dizziness following whiplash has used specific neck exercises involving motor relearning, strength, and endurance. The amount of effective practice for this type of therapy involves twice-a-week supervised exercise with a physiotherapist with home exercises for three months [46].

It is interesting to note that early after a concussion there is limited evidence related to stand-alone cervical, vestibular, or oculomotor therapies. However, if these domains are combined within physiotherapy, evidence demonstrates earlier recovery and improved outcomes [47]. This suggests that functional integration and variability in movement-based rehabilitation is preferable. Multimodal physiotherapy twice a week that involves a combination of cervical treatment, and vestibular and ocular retraining are some of the most effective treatments over the first month after a concussion [48], or weekly physiotherapy sessions with exercises over eight weeks [6].

Pushing cardiovascular autonomic adaptation with daily aerobic exercise, or eight sessions over 11 days has also been shown to be effective in accelerating recovery after concussion [49]. Currently, these types of training of cervical, ocular vestibular, and sub-threshold aerobic exercises represent best practices in early concussion interventions and require both supervised and specifically prescribed unsupervised practice.

A study investigating the effects of a pre-activity movement exercise intervention aimed to reduce the incidence of injury and concussion in rugby players. It showed a clear reduction in concussion incidence, with a dose-response effect if the exercises were increased from around two times per week to more than three times per week [50]. This suggests that regular exposure to exercises involving balance/perturbation, resistance, plyometric training, and sports-specific agility techniques may need a minimum practice dose if it is to be effective in practice. 

Practice prescription also needs to consider rest breaks to help with symptom control and fatigue and allow adequate time for consolidation of learning that requires rest and sleep [51]. Return to work and learn strategies use specific advice around breaks to help deal with photo/phono sensitivity, eye fatigue, and limitations in cognitive attention, memory, and concentration [52]. This is an important part of effective concussion rehabilitation.

Biomechanics

Biomechanics focuses on the physics and physiology of human movement. When considering the forces needed for concussion to occur, biomechanics plays a role in concussion prevention and the rehabilitation of normal biomechanical functions. Whiplash injury to the cervical spine is likely to co-occur yet cervical evaluation is often not included in standard concussion clinical assessments [53], where cervical and thoracic range of motion, palpation, strength, endurance, stability, and proprioceptive awareness are likely to be extremely important for assessment in general practice [54,55].

Brain injury prevention strategies should consider the linear and rotational head accelerations that lead to concussive brain injury [56]. Higher forces in areas of the brainstem nuclei involved in arousal are more likely to produce loss of consciousness [57]. Helmets may help dissipate large linear forces perhaps more relevant to severe TBI, and can protect skull fractures, facial injuries, lacerations, and cauliflower ears (common in rugby). However, helmets remain limited in their ability to significantly reduce the forces that lead to diffuse axonal injury in concussion. Not surprisingly, a study in Australian football has shown no effect of helmets in reducing concussions [58]. In American football, new soft-shell covers such as the ‘Guardian Cap’ are designed to reduce forces, with ongoing research underway [59-61].

Head and neck responses to internal and external perturbations have implications for concussion injury. Rugby players who had suffered a concussion within the past two years showed delayed anticipated head displacement and higher initial head acceleration. These alterations in motor control lead to biomechanical changes that place a vulnerable brain and brainstem at risk of further diffuse axonal damage [62]. Further understanding of the biomechanics of injury has implications for not only helmet and neck brace technologies but also training techniques for sporting skills such as tackling, heading soccer balls, and techniques for safely going to the ground. Techniques for falling and body position during collisions with other people or objects have relevance for not only sports and work but also fall prevention rehabilitation in the elderly, and accidental falls in children [63].

Increased trunk muscle size and thickness in multifidus and internal oblique muscles following concussion may indicate compensatory biomechanical strategies in movement and balance used to stiffen and stabilise the trunk [64-66]. Future research could expand on these findings to determine if these compensatory strategies are detrimental to long-term performance [66].

Post-traumatic benign paroxysmal positional vertigo (BPPV) is common after concussion and is successfully treated with canal repositioning manoeuvres that require specific biomechanical head movements in space to move debris within specific semi-circular canals back into the otolith organs [67]. BPPV is often effectively treated within one treatment session and should not be missed in the treatment of vertigo and balance following concussion [68].

Physical capacity

Too much rest following a concussion can be detrimental, and research on early physical activity and exercise now supports sub-threshold aerobic exercise as early as a few days following a concussion event. Controlled exercise protocols such as the Buffalo concussion treadmill test are a safe and reliable treatment to accelerate recovery [19]. Exercise protocols that are guided by symptom provocation, exertion levels, and heart rate response improve recovery [49], with the benefits possibly being driven by a number of important factors. Factors may include adaptation to abnormal autonomic cardiovascular responses common after concussion, and stimulating neuroprotective responses such as the release of neurotrophic factors important for structural brain recovery [49,69,70]. Early physical activity is also likely to have many psychological, physical, and social benefits that help build confidence, strength, and cardiovascular capacity that can be quickly lost with inactivity. In general practice, orthostatic intolerance testing of supine then standing blood pressure and heart rate helps screen for postural orthostatic tachycardia syndrome (POTS), a sign of autonomic dysregulation common after concussion [71]. POTS and autonomic dysfunction can be major contributors to persisting symptoms that should not be missed during assessment.

The association between neck strength and concussion incidence has been another area of interest due to its potential role in decreasing the impact forces on the brain, with a possible link when analysing current evidence [72,73]. Types of strength, stability, and anticipatory motor control needed to reduce the risk of concussion for certain functions and sports will be an important area of future research [62].

Attentional focus

While attention is an important part of the earlier principle of ‘cognitive selection and planning’, it is a component of training worth exploring further. Dual-task challenges of simultaneously completing cognitive attention tasks during motor performance is an area of great interest. Dual-task with tandem gait shows deterioration after concussion with slowed times [37]. This reflects a difficulty with balance and gait performance when attentional demand is required for simultaneously integrating other tasks. In competitive sports, these deficits may explain the increased risk of injury and repeat concussion [72] as attention has been previously linked to musculoskeletal injury [74]. Shifts in neural activity after concussion may indicate changes in sensory integration and attention, which may contribute to neuromuscular changes and increased injury risk [35]. While there is limited research evidence with regard to dual-task assessment and training, future concussion protocols could investigate gait velocity, cognitive errors, and movement performance quality to determine what measures will provide meaningful information to confirm concussion diagnosis and help facilitate recovery. Training protocols can incrementally introduce different types of attentional tasks that reflect the functions and tasks needed to reach a person’s required rehabilitation goals.

People with attention-deficit/hyperactivity disorder (ADHD) experience difficulties with attentional focus, and can present with symptoms mimicking concussion, which can make assessment interpretation difficult [75]. This becomes all the more challenging given that people with ADHD show deficits in vestibular-ocular motor testing of saccades, convergence, vestibular-ocular reflex, and visual motion sensitivity at baseline [76]. People with ADHD also show reduced scores on neurocognitive performance [77]. This lends support to the use of multimodal baseline concussion testing to help interpret post-concussion assessments. While attention is involved in many brain functions tested following concussion, any association of ADHD with concussion recovery outcomes is not yet clear [78].

Following concussion, anxiety and fear avoidance can theoretically contribute to an overlay of functional neurological disorder and somatic symptoms disorder [79]. Distraction techniques and a shift to more external attention demands are known to improve signs and symptoms in these disorders [80,81], which will have relevance in rehabilitation for concussion/TBI where functional overlay is suspected. As observed in many populations, internalising attention can be detrimental to performance, ranging from ‘choking’ in elite sports to fall risk in the elderly [82]. Currently, symptom reporting helps to determine readiness for return to play decision in sports; however, after more intense physically demanding tests, symptom provocation is more likely [83]. This raises questions about how much internal attentional focus on symptoms is needed to support clinical decisions, while also considering that an overemphasis on symptoms could potentially contribute to unhealthy illness behaviours in some people. The use of more diverse multimodal assessments that also include objective tests may have advantages with regard to individualised clinical decision-making [84].

Beliefs and self-efficacy

Mental health is an enormous concern following concussion. Anxiety and depression can easily be screened in general practice and are major issues post-brain injury [85,86], including serious mental health issues in children [87], with a two-fold increase in suicide risk [88]. However, the influence of concussion on negative beliefs extends beyond anxiety and depression. For some people, post-traumatic stress symptoms are a possibility and will need to be considered in the overall management [89,90]. Psychological assessments following concussion often classify findings into domains such as stress, anxiety, and depression; however, as evidence has shown with physical domains, combining cervical, vestibular, and ocular domains within treatment leads to better treatment outcomes [47]. More effective training strategies could consider how to combine psychological domains as well. ‘Psychological flexibility’ is one such combined mechanism that can influence concussion outcomes and involves strategies for handling distress and taking appropriate actions [91]. Targeting psychological flexibility early in rehabilitation has the potential to improve outcomes and is worth exploring.

The rise in research, media exposure, and delivery of community messaging can have the unintended consequences of creating negative beliefs about concussion at the level of both the individual and broader society. Over the past decade, we have witnessed a shift in the way concussions are viewed. Negative expectations about symptoms and recovery can lead to misattribution of normal symptoms, resulting in a ‘nocebo effect’ [92]. This needs to be seriously considered when communicating with concussion patients and their families during rehabilitation. More clear, accurate, and well-balanced messaging to the community about concussion recovery is also vital [92,93], especially given that both preinjury and post-concussion somatisation have been identified as risk factors for delayed recovery [94-96].

Summary

The 10 MTPs discussed above provide a comprehensive framework to plan and design individual concussion rehabilitation training for return to learn, work, and play with effective multidisciplinary working. The MTPs could be used to critically evaluate technologies that are rapidly emerging in this field. Virtual reality has the potential to explore combinations of physical, cognitive, and behavioural tasks that are more closely aligned with the demands of real life to help with both assessment and training following concussion [97]. Visual eye-tracking technologies also have the potential to assess many relevant vestibular, ocular, and cognitive functions relevant to concussion [98, 99]. Eye tracking has been used in vestibular practice for many years to assess peripheral and central dysfunction [100]; however, sports clinicians are less familiar with it and have had little access to or experience in the use of such devices [101]. Further education and collaboration regarding the potential benefits of these devices may help to institute specific individualised assessment and training protocols and may also help gain deeper insights into recovery mechanisms in relation to many of the MTPs. Eye tracking may also have the potential for remote tracking in concussion rehabilitation [102].

## Conclusions

Current evidence highlights the potential for effective interventions that target many different domains affected by concussions. There is a potential to improve recovery outcomes and recovery time and reduce the risk of secondary concussions, injury, and serious complications such as a decline in mental health, employment loss, and worsening long-term quality of life. Progress will continue to emerge from diverse fields including neurorehabilitation, sports, musculoskeletal, vestibular, exercise physiology, psychology, and neuropsychology practice. Given such vast diversity, it is anticipated that the MTPs discussed can provide a common language to facilitate future collaborations in both clinical care and research and guide practices that promote innovative, multidisciplinary rehabilitation. This may foster rapid innovations in this exciting area of practice.
